# Type I and III collagen contents and μ-calpain autolysis as a function of dry ageing time for eight different muscles from Hanwoo cattle

**DOI:** 10.5713/ab.24.0376

**Published:** 2024-10-25

**Authors:** Zhen Song, Inho Hwang

**Affiliations:** 1 College of Animal Science and Technology, Henan University of Science and Technology, Luoyang, China; 2 Department of Animal Science, Chonbuk National University, Jeonju, Korea

**Keywords:** Dry Ageing, Tenderness, Type I and III Collagen, μ-Calpain Autolysis

## Abstract

**Objective:**

Type I and III collagen content exert contrasting influences on meat tenderness. μ-calpain autolysis correlates with beef tenderness. Thus, the study aimed to determine the changes in these proteins.

**Methods:**

Three hundred twenty-four Hanwoo cattle, including cows and steers, and eight muscles were evaluated for proteolysis during dry ageing period. The ratios of type I and III collagen were determined by densitometric scans of bands resolved by sodium dodecyl sulfate (SDS)-polyacrylamide gel electrophoresis (PAGE), and μ-calpain activity was determined using casein zymography. Proteins involved in proteolysis were analysed by liquid chromatography-tandem mass spectrometry.

**Results:**

The ratio of type I and III collagen in every muscle showed a significant difference with increasing ageing times (p<0.05). In steers, the ratio decreased with increased ageing time, and in cows, except for Biceps femoris and Diaphragm muscles, a similar trend was observed. Significant differences in the ratio of type I and III collagen were found between different muscles of cows at the same ageing time (p<0.05), but no significant differences were found in steer muscles at the same ageing time (p>0.05). Casein zymogram results showed an inverse relationship between pH values and μ-calpain autolysis in every muscle. A significant reduction in μ-calpain activity was observed in all muscles with extended ageing times, while the rate of autolysis differed greatly (p<0.05) between muscles at the same ageing time. Interestingly, electropherogram analysis showed that cow muscles had a higher μ-calpain activity than steer muscles. Ageing time significantly influenced proteolysis, with 24 proteins showing marked changes.

**Conclusion:**

The ageing times significantly affect the ratio of type I and III collagen, coinciding with μ-calpain autolysis rates in steers. The ratio of type I and III collagen had a significant changes during the ageing period from cows, which may be related to the amount of collagen cross-linking.

## INTRODUCTION

Hanwoo cattle possess unique and valuable economic traits in terms of carcass and eating qualities, since they are primarily isolated within the Korean peninsula [1]. Hanwoo beef is regarded as one of the most expensive and high-quality types of beef compared to other cattle. Key features of Hanwoo beef include higher intramuscular fat (IMF) levels, less subcutaneous fat, thinner muscle fibers, and fewer connective tissues [2].

Several studies have found that tenderness is a more important factor in meat quality traits compared to juiciness and flavor [3]. Post-mortem ageing, the practice of hanging beef carcasses in a chilling room and allowing the meat to age for weeks or even months at controlled temperatures, relative humidity, and airflow [4] is known to improve beef tenderness, flavor, and aroma [5]. During this process, moisture is lost, and the fat becomes concentrated, increasing the fat-to-moisture ratio in dry-aged beef, which is believed to enhance the perception of juiciness [6]. As a result, dry ageing typically yields premium-quality meat.

Throughout the dry ageing period, muscles become more tender as proteins and lipids are broken down into smaller and more flavorful fragments by natural enzymes. Muscle proteolysis also contributes to meat tenderness [7]. The calpain proteolytic system is the major enzyme responsible for post-mortem muscle fiber degradation, which can improve the tenderness of bovine muscles [8]. μ-Calpain and m-calpain are the two main calpains found in beef [9], and calcium binding to calpains is necessary for proteolytic activation [10]. Previous studies have indicated that μ-calpain activity remains relatively stable during post-mortem ageing [11]. In contrast, other studies suggest that μ-calpain is the enzyme responsible for protein hydrolysis and meat tenderization during the post-mortem period [12].

Except for calpain, the proteolysis of collagen is also important during the post-mortem ageing period. In the animal body, collagen is the most abundant and major protein in connective tissues. Collagen provides tensile strength and elasticity in skeletal muscle, and it is associated with meat background toughness [13]. Although 26 different collagen types have been identified, collagen type I and III are the major types in animal muscle tissue. And type III collagen is positively correlated with meat tenderness [14]. Thus, the ratio of type I to type III collagen plays a critical role in determining meat tenderness.

Beef tenderness is crucial for manufacturers, as it is one of the key factors influencing consumers’ appreciation of meat. Additionally, the ubiquitous μ-calpain becomes active immediately after slaughter, leading to protein degradation and cell breakdown, which facilitates further proteolysis. Therefore, our objective was to evaluate the ratio of type I and III collagen, μ-calpain, and proteolysis from different dry ageing times (0 d, 20 d, 40 d, 60 d, 90 d), different muscles (*Longissimus Lumborum* [LL], *triceps brachii* [TB], *Biceps femoris* [BF], *Hind shank* [SN], *Diaphragm* [DP], *Supraspinatus* [SS], *Foreshank* [FS], and *Semimembranosus* [SM]), and different genders (cows and steers). The result will contribute to a better understanding of the relationships among calpain, collagen, proteolysis, and beef tenderness.

## MATERIALS AND METHODS

### Carcass selection

Three hundred and twenty-four Hanwoo cattle cows (n = 180), and steers (n = 144) were sampled from the commercial meat processing plant. They were slaughtered at different ages (Tables 1, 2). The animals were transported 100 to 200 km to a commercial abattoir and held overnight, with access to water but no feed, prior to slaughter.

### Sample preparation

Six muscles (LL, TB, BF, SN, DP, and SS) were collected from cow carcasses, and four muscles (BF, TB, FS, and SM) were collected from steer carcasses. The muscles were aged under natural chiller drying conditions. The procedure included three distinct ageing circumstances: (i) All muscles aged at 2°C and 65% air humidity at 0 d and 20 d; (ii) All muscles aged at 2°C and 75% air humidity at 40 d; and (iii) All muscles aged at 4°C and 85% air humidity at 60 d and 90 d. Each muscle type contained 6 samples for each ageing time of cows and 9 samples for each ageing time of steers. When the ageing time was completed, cover fat and dried outside skin were removed. The rest muscle tissues were cut into 3 parts, and each part of more than 5 g was vacuum-packaged and stored at −80°C until analysis of the proteolysis of collagen, calpain, and sodium dodecyl sulfate (SDS)-polyacrylamide gel electrophoresis (PAGE).

### Collagen type parameters

Type I and type III collagen were extracted using acetic acid and pepsin. The key steps of the method were as follows: 4 g of cut muscle samples were mixed with 24 mL of 0.5 M acetic acid and homogenized at 9,400×g for 15 s per cycle, repeated four times using an Ultra Turrax T25 (IKA Labortechnik; Jkika Works (Asia) Sdn., Bhd., Rawang, Malaysia). Next, 1.33 g of pepsin was added to each sample, and the mixture was stirred in a chilled room for 48 h. The supernatant was collected after centrifugation at 10,000×g, for 20 min at 4°C. Samples were salted with 1M NaCl and then centrifuged again at 15,000×g, for 15 min at 4°C. To dissolve the pellets, 8mL of 1 M acetic acid solution was used per gram of pellet. The dissolved pellets were then dialyzed against 0.1% acetic acid, ensuring that the volume ratio between the sample and dialyzing buffer exceeded 100:1. The concentration of each sample was adjusted to 0.5 mg/mL using sample buffer (0.125 M Tris-HCl, pH 6.8, containing 4% SDS and 20% glycerol). SDS-PAGE was carried out following the method of Laemmli [15]. Five μg of protein was loaded into each well, and electrophoresis was performed at a constant voltage of 100 V for 2 h using a 6% separating gel and a 4% stacking gel. The gel was stained overnight with Coomassie Brilliant Blue R-250 in 5% (v/v) methanol and 7.5% (v/v) acetic acid, then destained for 3 h with fresh solution changes every 30 min using 7.5% (v/v) acetic acid and 25% (v/v) methanol. Photographs were taken using a VersaDOC (Bio-Rad, Hercules, CA, USA) system with Quantity One software.

For the western blot, protein transfer was conducted at a constant 200 mA for 16 h in a chilled room. The membrane was blocked with 5% non-fat milk, and the primary antibodies (1:2000 for Type I collagen and 1:5,000 for Type III collagen) were incubated for 1 hour at room temperature (RT). The membrane was rinsed three times with tris buffered saline with Tween (TBST) (50 mM Tris-HCl, PH 8.0, 150 mM NaCl, and 0.1% Tween-20) for 5 min each. The secondary antibody (1:20,000) was then incubated for 1 h at RT, followed by three washes with TBST for 5 min each. One mL of solution A and 1 mL of solution B from the ECL Select Western Blotting Detection Reagent (GE, Washington, DC, USA) were added for 1 min at RT. Photographs were taken using the VersaDOC (Bio-Rad) system with Quantity One software.

### μ-Calpain parameters

The cut samples (3 g) were homogenized with 100 mM Tris and 10 mM ethylenediaminetetraacetic acid disodium salt, pH 8.3, at 17,000×g for 15 s/time, 3 times using an Ultra Turrax T25. The supernatant was collected after centrifugation at 8,800×g for 30 min at 4°C. Casein zymography was performed following the procedures of Veiseth et al [16]. The supernatant was loaded in each well of a large gel (22.3 cm×20 cm, 1.0 mm thick, 12.5% separating gel). Electrophoresis was conducted at 100 V for 16 h at 4°C, followed by incubation with 20 μM CaCl_2_ to activate μ-calpain. Before incubation (16 h), the gels were washed for 20 min, 3 times with incubation buffer. The gel was then stained for 2 h using Coomassie Brilliant Blue R-250 in 5% (v/v) methanol and 2% (v/v) acetic acid, destained for 2 h with a solution change every 30 min using 7% (v/v) acetic acid and 20% (v/v) methanol, and a photograph was taken with VersaDOC (Bio-Rad) machine’s Quantity One software.

A Western blot transfer was conducted at 300 mA for 2 h in a chilled room. The blocking buffer contained 5% non-fat milk in TBST. The primary antibody (1:1,250) was incubated for 1 h at RT, and the membrane was rinsed with TBST for 5 min, three times. The secondary antibody (1:20,000) was incubated for 1 h at RT followed by three 5-minute washes with TBST. The ECL Select Western Blotting Detection Reagent was applied for 1 min at RT, and photographs were taken using a VersaDOC (Bio-Rad) machine with Quantity One software.

### Proteolysis analysis

Three grams of cut muscle samples were homogenized with 9 mL of extraction buffer at 9,400×g for 30 s, repeated twice, using an Ultra Turrax T25 (IKA Labortechnik, Jkika Works (Asia) Sdn., Bhd.). Two milliliters of supernatant were collected after centrifugation at 10,000×g for 20 min at 4°C. The supernatant was diluted to 20-fold, and the protein concentration was measured using the DCTM Protein Assay Reagent A and B (Bio-Rad). The concentration of each sample was adjusted to 2 mg/mL with sample buffer (0.125 M Tris-HCl, pH 6.8, containing 4% SDS, 20% glycerol, and 10% 2-Mercaptoethanol), and 30 μg of protein was loaded into each well. SDS-PAGE was performed according to the method of Laemmli using the Bio-Rad system [15]. For proteome sequencing a slight modification was made to the SDS-PAGE procedure. A total of 125 μg of protein was loaded into each well (on a large gel, 22.3 cm×20 cm, 1.0 mm thick). Electrophoresis was carried out at a constant 100 V for 16 h at 4°C. After electrophoresis, the gel was stained with 0.08% Coomassie Brilliant Blue G-250 in 1.6% (v/v) ortho-phosphoric acid, 8% (v/v) ammonium sulfate, and 20% (v/v) ethanol overnight, then destained with deionized water for about 2 h changing the water every 30 min. The gel was photographed, and the sequencing band was analyzed using a VersaDOC (Bio-Rad) machine with Quantity One software. The liquid chromatography-tandem mass spectrometry (LC-MS/MS) procedure, commissioned by center for University-Wide Research Facilities of Jeonbuk National University, was used to analyze the proteins.

### Statistical analysis

The ratio of type I and III collagen as determined by densitometric scans of band resolved by SDS-PAGE using a quantity one, and μ-calpain activity from casein zymography was analyzed using Image Lab (Bio-Rad,). All data was analyzed using a General Linear Model Procedure of the SAS version 9.3 program with random effect of animals (SAS Institute, Cary, NC, USA). The significance level was estimated at p<0.05.

## RESULTS AND DISCUSSION

### Changes in collagen type I and III during dry ageing

**Figure 1:**
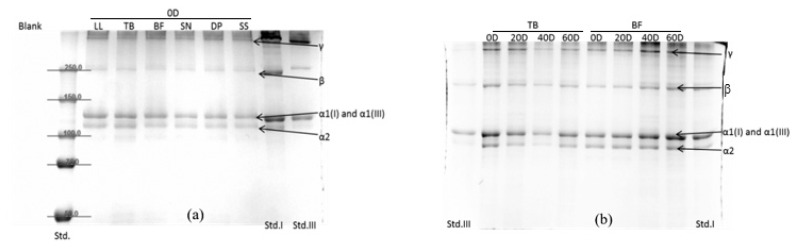
Collagen type I and type III of different aged muscles from Hanwoo cattle by SDS-PAGE method. (a) Collagen type I and type III from different muscles of cows at the same ageing time; (b) TB and BF muscles with different ageing time of steers. SDS-PAGE, sodium dodecyl sulfate polyacrylamide gel electrophoresis; LL, *Longissimus lumborum*; TB, *Triceps brachii*; BF, *Biceps femoris*; SN, *Hind shank*; DP, *Diaphragm*; SS, *supraspinatus*. Lane Std I, the standard of type I collagen; lane Std III, the standard of type III collagen.

Meat tenderness is a major factor in meat quality, and connective tissue (primarily collagen matrix) contributes to background toughness [17]. Previous studies [6], along with our proteome profile (Table 3), have demonstrated that while degradation of myofibrillar components is a key mechanism during chiller ageing, the connective tissue remains relatively stable. Studies have also indicated that the content of type I and III collagen is related to meat tenderness [18]. In this study, we aimed to quantify changes in type I and III collagen during chiller ageing across different muscles using SDS-PAGE (Figure 1). Our gel data indicated that collagen from different ageing times and genders contained two prominent chains (125 kDa and 115 kDa), as well as β and γ components. The 125 kDa and 115 kDa bands correspond to α chains from type I and III collagen, as confirmed by western blotting. No smaller components lower than 115 kDa were observed in the electropherograms, suggesting that the extracted collagen was not further degraded during the ageing process. The β and γ chains, representing cross-linked collagen molecules, showed lower band intensities than the α chains on the electrophoresis map.

**Figure 2:**
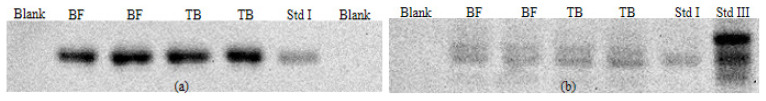
Western blot patterns for collagen type I and type III. (a) Type I collagen western blot; (b) type III collagen western blot. BF, *Biceps femoris*; TB, *triceps brachii*. Lane Std I, the standard of type I collagen; lane Std III, the standard of type III collagen.

The most prevalent and thoroughly researched collagen is type I collagen. It is the primary collagen of tendons, skin, ligaments, and cornea and makes up more than 90% of the organic mass of bone. Two identical α_1_ chains and one α_2_ chain often form a hetero-trimer to form the type I collagen triple helix. Meanwhile, type III collagen has three α_1_ (III) chains. Thus, we used Western blot to identify the purified products. Western blot photograms indicated that the band of α1 contained type I and III collagen (Figure 2). The western blot of type III collagen had two bands in the figure. The reason may be that collagen type III contained the intramolecular disulfide bond. Native disulfide bond formation is important for non-covalent interactions and the stable native product [19]. These results suggest that the method of collagen purification could help type III collagen hold the intramolecular disulfide bond for all muscles in this study. This study used the density of α_1_ and α_2_ bands to get the relative concentration of type I and type III collagen.

The type I and III collagen baseline characteristics of beef in this study are summarized in Tables 1, 2. Results showed that the ratio of type I and III collagen decreased with the ageing time extension of steers, but the ratio of type I and III collagen from cows did not show the same trend, this can be attributed to the collagen cross-linking which increases with age, and after 30-month collagen solubility had the lowest value in beef [20]. The cows ages over 30-month, thus, different muscles from cows had different amounts of collagen cross-linking. Collagen cross-linking broke and collagen hydrolyzed as ageing time was extended. However, the ratio of type I and III collagen had the lowest values (90 d) in all samples from cows, and it is widely known that beef tenderness increase with the increase of the aging time. These findings highlight the significant role of type I collagen in beef tenderness through collagen transformations. The ratio of type I and III collagen had a significant difference at the same ageing time among different muscles (p<0.05) from cows and the same muscle also had a significant difference among the different ageing times (p<0.05) (Table 1). Results suggested that different muscle contained different contents of collagen, and type I and III collagen cross-linking broke maybe had a different rate.

The order of the ratio of type I and III collagen from cows is SN>DP>LL>SS>TB>BF at 0 d ageing time. SN muscles had the highest values of the ratio of collagen type I and III in every ageing time in all muscles. This can be attributed to SN containing a lot of muscles such as *Peroneus tertius*, *Extensor digitorum medius*, *Extensor digitorum longus*, *Extensor digitorum lateralis*, *Peroneus longus*, *Flexor digitorum profundus*, and *Soleus*. Since tendons, which are rich in type I collagen are present between these muscles, SN had the highest ratio of type I to III collagen in all cow muscles. At 0 d of ageing, the BF and TB muscles in cows exhibited a lower ratio of type I and III collagen compared to other muscles, likely due to differences in collagen cross-linking between muscles [21]. As ageing progressed, the ratio of type I to III collagen in DP muscles decreased by 58.2% from 0 d to 20 d, showing the largest decrease, which suggests the highest tenderness among all muscles. Conversely, the ratio of type I to III collagen in BF muscles improved at the ageing time of 40 d (p<0.05), indicating that BF muscles had a large amount of cross-linking collagen was broken.

For the steers, the ratio of type I and III collagen had no statistical difference among different muscles at every ageing time and the values of the ratio of type I and III collagen decreased in all the muscles with ageing time extension. With an aging time, extension to 60 d, the ratio of type I and III collagen had the lowest values, suggesting the tenderness is higher than on other ageing days. The ratio of type I and III collagen in cows and steers showed a different trend. These differences can be ascribed that cows and steers had different ages in this study, and the amount of collagen cross-linking increased as age increased, particularly after 30-month collagen solubility had the lowest value in beef [20].

The total content of collagen, especially, type I collagen was negatively correlated with meat tenderness. Results showed that the ratio of type I and III collagen had a different trend between cows and steers, the values from steers decreased with the ageing time extension, but the values from cows had improved in BF and TB muscles at the ageing time of 40 d and 60 d, and why the tenderness could not decrease, the reason might be that the content of soluble collagen was more important related to beef tenderness than total collagen during the post-mortem ageing time, and higher soluble collagen with higher beef tenderness [22]. The ratio of type I and III collagen decreased, and cows and steers all had the lowest values at the final ageing time day. These can be ascribed that during post-mortem ageing time, the collagen fibrils were separated from the endomysium and were broken down into small peptides and collagen was hydrolyzed which could improve beef tenderness [23,24]. Age can be affected the collagen cross-linking as discussed earlier. Different muscles had different degradation rates of the ratio of type I and III collagen from cows during ageing times, the ratio of type I and III collagen of LL SN and SS decreased with the increase of the ageing time, but BF DP and TB increased after an initial decrease. Results suggested that BF DP and TB muscles had higher content of type I collagen cross-linking than type III collagen cross-linking after 30 months of age, and age had little effect on the amount of collagen cross-linking in LL SN and SS muscles. Results suggest that different muscles had different collagen cross-linking values [21].

### Calpain activity and Proteome profiles of myofibrils as a function of dry ageing days

Although multiple factors influence meat tenderness, the calpain proteolytic system (including μ-calpain, m-calpain, and calpastatin) is the primary contributor to meat tenderness [11,25]. Previous studies have shown that while m-calpain does not degrade with extended ageing [11], μ-calpain proteolysis can serve as an indicator of proteolytic activation in postmortem muscle [12], and hydrolyzed fragments of type I collagen can enhance μ-calpain activity [26]. Therefore, μ-calpain was the focus of in this study.

**Figure 3:**
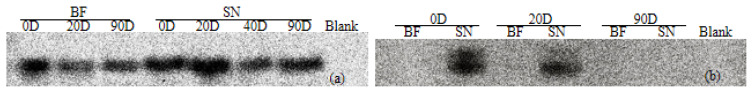
Western blot patterns for μ-calpain of different muscles and different ageing times. (a) Western blot of μ-calpain with SDS-PAGE; (b) western blot of μ-calpain with non-denaturing PAGE. SDS-PAGE, sodium dodecyl sulfate polyacrylamide gel electrophoresis; BF, *Biceps femoris*; SN, *Hind shank*.

Western blot analysis using SDS-PAGE indicated that a single μ-calpain band at 80 kDa across different muscles over the ageing period (Figure 3A), consistent with previous findings [27]. Further analysis with non-denaturing PAGE revealed that μ-calpain activity decreased as ageing progressed (Figure 3B), likely due to μ-calpain undergoing extensive autolysis upon activation [28].

**Figure 4:**
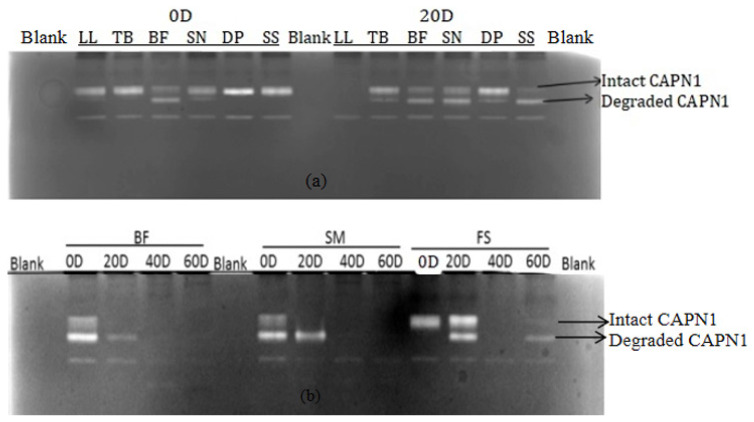
μ-Calpain activity in different muscles and different ageing times from Hanwoo cattle by using Casein Zymography method. (a) μ-Calpain activity of different muscles at different ageing times from Hanwoo cows; (b) μ-calpain activity of different muscles at different ageing times from Hanwoo steers. LL, *Longissimus lumborum*; TB, *Triceps brachii*; BF, *Biceps femoris*; SN, *Hind shank*; DP, *Diaphragm*; SS, *supraspinatus*; SM, *Semimembranosus*; FS, *Foreshank*.

Casein zymography demonstrated that μ-calpain activity varied across different muscles and decreased with extended ageing (Figure 4). This technique was also used to assess the degradation rate of μ-calpain across different muscles and ageing times (Tables 1, 2). The results showed that μ-calpain activity in cows followed the order: DP>SN> SS>TB>LL>BF. In steers, the order was TB>FS>SM>BF. In addition, the study found a negative correlation between pH levels and μ-calpain autolysis in postmortem animals. Our findings are consistent with prior studies, which showed that postmortem pH decline influence influences μ-calpain activity, with lower pH leading to earlier autolysis [29]. Significant differences (p<0.01 or p<0.001) were observed in μ-calpain activity (both intact and degraded) across different ageing periods. These results support the conclusion that μ-calpain proteolysis enhances meat tenderness [11,12]. Moreover, steers exhibited lower μ-calpain activity than cows at 0 days of ageing, suggesting higher μ-calpain autolysis in steers (Tables 1, 2). This aligns with findings that steers tend to have greater tenderness than cows, and younger animals generally exhibit higher tenderness than older animals [30]. In cows, collagen shows more cross-linking than in steers. Both the proportion of type I collagen and μ-calpain autolysis in steers followed a similar trend, with values decreasing over the ageing period. This may be because hydrolyzed fragments of type I collagen can enhance μ-calpain hydrolysis [26].

**Figure 5:**
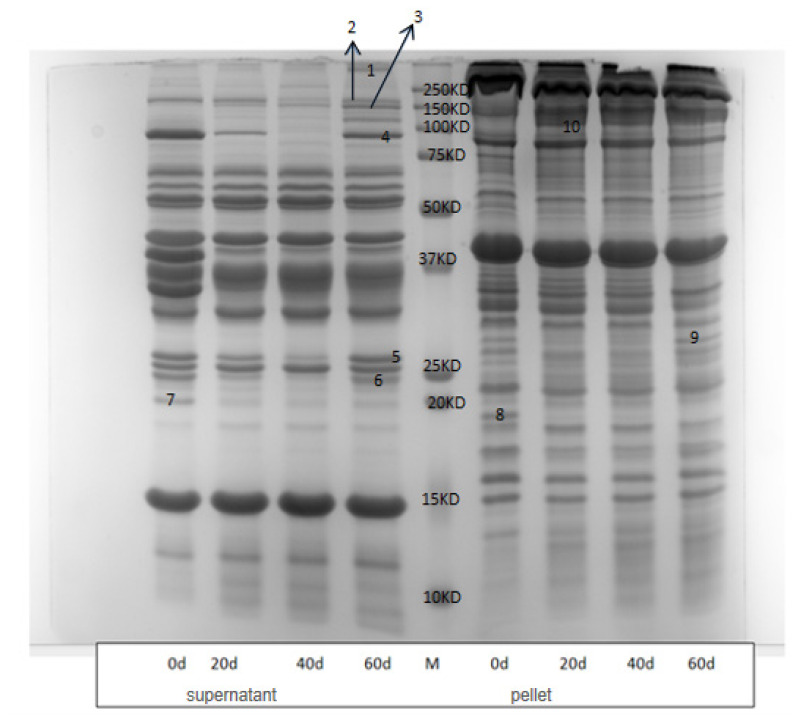
SDS-PAGE profile of semimembranosus muscle for Hanwoo steer at aged 0, 20, 40 and 60 days. Lane M, molecular weight marker. Number 1 to 10 means sequencing band number and identified bands are tabulated in Table 3. M, standard of marker; SDS-PAGE, sodium dodecyl sulfate polyacrylamide gel electrophoresis.

Ageing can disrupt collagen cross-linking, increase myofibril fragmentation, and dissolve myofibrillar proteins. The calpain proteolytic system is the primary enzyme system responsible for post-mortem muscle fiber degradation. Therefore, we used SDS-PAGE to detecting proteolytic degradation in post-mortem muscles. Our SDS-PAGE results revealed that several protein and/or peptide bands disappeared with extended ageing, while some of bands showed a gradual increase with ageing time. Previous studies suggest that calpain mediates proteolysis during the post-mortem ageing time [31]. Consequently, we selected SM muscle for comparison of post-mortem proteolysis across different ageing times. This selection was based on its higher μ-calpain autolysis compared to BF and TB muscles. Casein zymography showed significant differences (p<0.001) in μ-calpain activity (both intact and degraded) among different ageing periods. The proteolytic products during ageing were separated using SDS-PAGE (Figure 5) and identified by LC/MS/MS, as recommended by previous studies on post-mortem muscles [32].

Twenty-four proteins (Myomesin-1, K10, K17, K73, K78, K40, Glycogen phosphorylase, Alpha-actinin-2, CA-III, NAP-22, Adiponectin, TIM, GSTM1, AK1, DJ-1, Peroxiredoxin-1, Peroxiredoxin-2, VDAC-3, MLC1/MLC3, Myozenin-1, Myosin-1, Myosin-2, Myosin-7) were identified from 10 bands that changed with ageing times (Table 3). Proteins can migrate to the same band when denatured by SDS, complicating definitive identification of individual proteins. These proteins were identified from 10 bands with significant differences as ageing time increased (p<0.001) (Table 3). These findings suggest that the identified proteins are likely involved in post-mortem proteolysis. For instance, keratins may be related to the dissolution of myofibrils, while changes in Alpha-actinin-2 might be linked to cellular structural destruction. Our results align with studies that associate keratins [33], alpha-actinin [34], myozenin-1, myosin-2 [35], glycogen phosphorylase [36], carbonic anhydrase 3, myosin-1, myosin-7, and CA-III [37], DJ-1 and peroxiredoxin [38], and adiponectin [39] with beef tenderness. In addition, we observed hydrolysis of NAP-22, TIM, GSTM1 and VDAC-3 proteins. These proteins could serve as potential markers for selecting beef tenderness.

## CONCLUSION

The current data revealed that dry ageing significantly affects the content of type I and type III collagen, with the ratio of type I to type III collagen decreasing as ageing time increases (excluding muscles with cross-linked collagen). This decrease aligns with the rate of μ-calpain autolysis. The study identified 24 proteins that changed during the ageing period. Furthermore, our Western blot analysis showed that the α1 chains of type I and type III collagen from meat tissues have the same mobilities on SDS-PAGE.

## Figures and Tables

**Table 1:** Proteolysis of different ageing time and different muscle types of cows

Muscles	Treatments^1)^	0 d	20 d	40 d	60 d	90 d	SEM	F-value
BF	Months	59.00	47.67	47.67	41.33	48.67	11.29	0.94
	pH	5.59^ab^	5.39^Zd^	5.65^Ya^	5.57^Ybc^	5.52^c^	0.10	17.69^***^
	TypeI	51.81	68.40	62.10	50.89	55.95	11.05	1.56
	TypeIII	22.24^ab^	20.93^ab^	14.16^b^	18.86^ab^	27.40^a^	6.26	2.12
	Ratio	2.38^Yb^	3.26^YZab^	4.61^Xa^	2.70^XYb^	2.05^XYb^	1.11	5.19^*^
	μ-Calpain1	70.13^Ya^	14.40^Zb^	-^Y^	-	-	39.4	9.99^**^
	μ-Calpain2	29.87^X^	52.27	-^Y^	33.33	-	12.1	1.17
DP	Months	59.00	47.67	47.67	41.33	48.67	11.29	0.94
	pH	5.63	5.59^X^	5.81^X^	5.73^X^	5.59	0.13	2.13
	TypeI	85.30^a^	57.80^b^	57.90^b^	53.07^b^	53.92^b^	13.29	24.06^***^
	TypeIII	14.28^c^	23.13^b^	19.57^bc^	24.27^b^	36.45^a^	7.57	12.04^**^
	Ratio	5.98^Xa^	2.50^Zbc^	3.19^XYb^	2.22^Ybc^	1.49^Yc^	1.68	21.75^***^
	μ-Calpain1	100.00^Xa^	86.00^Xa^	56.30^Xb^	24.83^c^	-	33.40	30.02^***^
	μ-Calpain2	-^Y^	14.00	43.97^X^	41.83	33.33	32.10	1.07
LL	Months	59.00	47.67	47.67	41.33	48.67	11.29	0.94
	pH	5.54^b^	5.44^YZc^	5.61^Ya^	5.57^Yab^	5.53^b^	0.06	9.84^**^
	TypeI	63.23	66.00	54.67	61.80	57.00	11.26	0.39
	TypeIII	12.49^c^	13.98^c^	14.19^c^	23.23^b^	29.05^a^	6.65	17.58^***^
	Ratio	5.17^XYa^	4.83^XYa^	3.84X^Yab^	2.66^XYb^	1.96^XYb^	1.45	5.61^*^
	μ-Calpain1	97.53^Xa^	14.67^Zb^	-^Y^	-	-	58.59	40.75^***^
	μ-Calpain2	2.47^Y^	18.67	-^Y^	-	-	11.46	0.94
SN	Months	59.00	47.67	47.67	41.33	48.67	11.29	0.94
	pH	5.56^bc^	5.52^XYc^	5.74^XYa^	5.65^XYab^	5.57^bc^	0.09	8.12^**^
	TypeI	79.00	65.50	66.70	62.80	72.40	9.17	1.84
	TypeIII	11.08^b^	14.10^ab^	13.99^ab^	19.00^ab^	27.15^a^	7.76	2.01
	Ratio	7.25^Xa^	5.08^Xab^	5.01^XYab^	4.10^Xab^	2.67^Xb^	1.96	2.99
	μ-Calpain1	96.07^Xa^	51.20^Yb^	17.17^Yc^	13.80^c^	-	38.27	17.94^***^
	μ-Calpain2	3.93^Yb^	48.80^a^	86.20^Xa^	49.50^a^	-	33.67	6.77^**^
SS	Months	59.00	47.67	47.67	41.33	48.67	11.29	0.96
	pH	5.60^bc^	5.52^XYc^	5.74^XYa^	5.62^XYb^	5.59^bc^	0.08	9.82^**^
	TypeI	67.50	63.10	63.47	48.40	50.90	10.91	2.38
	TypeIII	15.17^b^	13.79^b^	16.40^b^	22.70^b^	32.30^a^	7.48	6.64^**^
	Ratio	5.06^XYa^	4.61^XYab^	3.93^XYabc^	2.21^Ybc^	1.58^Yc^	1.71	3.77^*^
	μ-Calpain1	90.03^XYa^	16.63^Zb^	17.77^Yb^	11.53^b^	-	37.46	9.68^**^
	μ-Calpain2	9.97^XY^	50.37	48.90	21.80	-	22.74	1.25
TB	Months	59.00	47.67	47.67	41.33	48.67	11.29	0.94
	pH	5.54	5.55^X^	5.64^Y^	5.63^XY^	5.55	0.07	2.08
	TypeI	57.41	51.80	60.30	64.90	57.75	12.76	0.55
	TypeIII	14.43^c^	20.66^ab^	25.19^a^	15.70^bc^	26.33^a^	4.86	8.19^**^
	Ratio	4.14^XY^	2.51^Z^	2.39^Y^	4.10^X^	2.19^XY^	1.25	2.45
	μ-Calpain1	100.00^Xa^	70.58^XYb^	15.40^Yc^	-	-	42.95	58.32^***^
	μ-Calpain2	-^Y^	29.43	84.43^X^	-	-	42.86	37.92^***^

1) Type I, relative quantitative percentage of the band (α1 of type I collagen) in the electrophoretogram; Type III, relative quantitative percentage of the band (α1 of type III collagen) in the electrophoretogram; ratio, the relative content of type I/III collagen in the electrophoretogram; μ-Calpain1, intact μ-calpain (80 kDa); values in the table means the relative content between 80 kDa and 78 kDa; μ-Calpain2, degraded μ-calpain (78 kDa); values in the table means the relative content between 80 kDa and 78 kDa.

a,b Means within row with different superscripts are significantly different of the same variable;

X,Y means within column with different superscripts are significantly different of the same variable.

* p<0.05,

** p<0.01,

*** p<0.001.

SEM, standard error of the mean; BF, *Biceps femoris*; -, not found; DP, *Diaphragm*; LL, *Longissimus lumborum*; SN, *Hind shank*; SS, *Supraspinatus*; TB, *Triceps brachii*.

**Table 2:** Proteolysis of different ageing time and different muscle types of steers.

Muscles	Treatments^1)^	0 d	20 d	40 d	60 d	SEM	F-value
BF	Month	28.33	29.25	28.67	29.57	1.86	0.69
	pH	5.50^Y^	5.46^Y^	5.50^Y^	5.52^Y^	0.07	1.20
	Type I	57.97^a^	56.4^Xa^	50.45^XYab^	42.47^b^	12.32	2.96
	Type III	23.11^c^	24.10^bc^	33.47^ab^	38.69^a^	10.70	5.44^**^
	Ratio	2.94^a^	2.66^a^	1.69^ab^	1.23^b^	1.30	3.68^*^
	μ-Calpain1	43.00^Za^	3.54^Zb^	-^b^	-^b^	24.89	13.67^***^
	μ-Calpain2	57.13^Xa^	58.96^XYa^	-^b^	-^b^	40.85	9.1^***^
FS	Month	28.67	29.25	28.67	29.57	1.88	0.39
	pH	5.59^XY^	5.52^XY^	5.53^XY^	5.56^XY^	0.11	0.79
	Type I	58.03^a^	45.53^XYb^	48.45^XYab^	45.77^b^	11.03	2.88
	Type III	22.77^b^	31.29^ab^	30.17^ab^	35.61^a^	9.35	3.29^*^
	Ratio	3.17^a^	1.66^b^	1.65^b^	1.42^b^	1.32	4.08^*^
	μ-Calpain1	85.24^Xa^	24.21^Yb^	9.92^bc^	-^c^	40.07	29.41^***^
	μ-Calpain2	14.76^Zb^	63.09^XYa^	23.40^ab^	28.57^ab^	39.48	2.76
SM	Month	29.33	29.25	28.67	29.57	2.08	0.20
	pH	5.52^Y^	5.50^XY^	5.51^Y^	5.50^Y^	0.09	0.54
	Type I	48.63	41.77^Y^	40.06^Y^	34.67	12.81	1.74
	Type III	22.74^b^	26.68^ab^	33.77^a^	36.21^a^	10.20	3.66^*^
	Ratio	2.95^a^	1.763^ab^	1.36^ab^	0.99^b^	1.75	2.12
	μ-Calpain1	50.44^YZa^	3.66Z^b^	-^b^	-^b^	28.15	16.99^***^
	μ-Calpain2	49.56^XYab^	83.84^Xa^	33.33^bc^	-^c^	44.07	8.10^***^
TB	Month	28.67	28.66	29.25	29.57	1.88	0.39
	pH	5.70^X^	5.59^X^	5.60^X^	5.62^X^	0.14	1.34
	Type I	61.43^a^	50.28^XYab^	51.10^Xa^	39.17^b^	12.89	5.95^**^
	Type III	21.98^b^	27.31^b^	29.42^b^	42.01^a^	12.28	5.22^**^
	Ratio	3.59^a^	2.08^ab^	2.00^ab^	1.08^b^	1.77	3.52^*^
	μ-Calpain1	74.03^XYa^	60.80^Xa^	11.03^b^	-^b^	36.24	32.7^***^
	μ-Calpain2	25.97^YZa^	39.30^Ya^	22.30^ab^	-^b^	25.17	4.11^*^

1) Type I, Relative quantitative percentage of the band (α1 of collagen type I) in the electrophoretogram; Type III, Relative quantitative percentage of the band (α1 of collagen type III) in the electrophoretogram; Ratio, the relative content of collagen type I/III; μ-Calpain1, intact μ-calpain (80 kDa); values in the table means the relative content between 80 kDa and 78 kDa; μ-Calpain2, degraded μ-calpain (78kDa); values in the table means the relative content between 80 kDa and 78 kDa.

a,b Means within row with different superscripts are significantly different of the same variable;

X,Y means within column with different superscripts are significantly different of the same variable.

* p<0.05,

** p<0.01,

*** p<0.001.

SEM, standard error of the mean; BF, *Biceps femoris*; -, not found; FS, *Foreshank*; SM, *Semimembranosus*; TB, *Triceps brachii*.

**Table 3:** Effect of ageing time on the change of SDS-PAGE band density, and identification of corresponding proteins

Band	Ageing				Identified fragment	Swiss-Prot number	MW of intact protein	F-value

	0	20	40	60				
1	0^b^	8.33^b^	11.8^b^	76.5^a^	Myomesin-1	P80473.1	37,282	23.85^***^
					K10	P06394.1	54,815	
					K17	A1L595.1	48,682	
					K73	A7YWK3.1	58,798	
					K78	A6QNX5.1	57,428	
2	31.8^a^	30.8^a^	15.1^c^	22.3^b^	Myomesin-1	P80473.1	37,282	23.97^***^
					K10	P06394.1	54,815	
					K17	A1L595.1	48,682	
					K73	A7YWK3.1	58,798	
					K78	A6QNX5.1	57,428	
3	15.6^a^	23.7^b^	19.7^b^	40.8^b^	Myomesin-1	P80473.1	37,282	18.09^***^
					K40	A7YWM2.1	47,789	
4	50.6^a^	16^c^	0^d^	33.4^b^	Glycogen phosphorylase	P79334.3	97,231	177.83^***^
					Alpha-actinin-2	Q3ZC55.1	103,713	
5	34^a^	22.6^c^	14^d^	29.5^b^	CA-III	Q3SZX4.3	29,351	58.21^***^
					NAP-22	P80724.3	22,997	
					Adiponectin	Q3Y5Z3.1	26,116	
6	41.4^a^	25.7^c^	0^d^	32.9^b^	TIM	Q5E956.3	26,672	120.14^***^
					GSTM1	Q9N0V4.3	25,618	
					CA III	Q3SZX4.3	29,351	
7	100^a^	0^b^	0^b^	0^b^	AK 1	P00570.2	21,650	Infinity^***^
					DJ-1	Q5E946.1	20,022	
					Peroxiredoxin-1	Q5E947.1	22,195	
					Peroxiredoxin-2	Q9BGI3.1	21,932	
8	100^a^	0^b^	0^b^	0^b^	MLC1/MLC3	A0JNJ5.1	20,918	Infinity^***^
9	36.1^b^	0^c^	0^c^	63.9^a^	VDAC-3	Q9MZ13.1	30,719	755.76^***^
					MLC1/MLC3	A0JNJ5.1	20,918	
					Myozenin-1	Q8SQ24.1	31,653	
					CA-III	Q3SZX4.3	29,351	
10	0^b^	34.3^a^	37.2^a^	28.5^a^	Myosin-1	Q9BE40.2	222,850	42.41^***^
					Myosin-2	Q9BE41.1	223,179	
					Myosin-7	Q9BE39.1	223,089	
df:3/11								

a–d Means of band density in arbitrary unit bearing different letter are significantly different within row.

*** p<0.001.

SDS-PAGE, sodium dodecyl sulfate polyacrylamide gel electrophoresis; MW, molecular weight; K10, keratin, type I cytoskeletal 10; K17, keratin, type I cytoskeletal 17; K73, keratin, type II cytoskeletal 73; K78, keratin, type II cytoskeletal 78; K40, keratin, type I cytoskeletal 40; CA-III, carbonic anhydrase 3; NAP-22, brain acid soluble protein 1/neuronal axonal membrane protein NAP-22; TIM, triosephosphate isomerase; GSTM1, glutathione S-transferase Mu 1; AK 1, adenylate kinase isoenzyme 1; DJ-1, protein/nucleic acid deglycase DJ-1; VDAC-3, voltage-dependent anion-selective channel protein 3; MLC1/MLC3, myosin light chain 1/3.
